# How Can Nutrition Research Better Reflect the Relationship Between Wasting and Stunting in Children? Learnings from the Wasting and Stunting Project

**DOI:** 10.1093/jn/nxac091

**Published:** 2022-06-10

**Authors:** Kate Sadler, Philip T James, Zulfiqar A Bhutta, André Briend, Sheila Isanaka, Andrew Mertens, Mark Myatt, Kieran S O'Brien, Patrick Webb, Tanya Khara, Jonathan C Wells

**Affiliations:** Emergency Nutrition Network, Kidlington, United Kingdom; Emergency Nutrition Network, Kidlington, United Kingdom; Center for Global Child Health, Hospital for Sick Children, Toronto, Canada; Center of Excellence in Women & Child Health, The Aga Khan University, Karachi, Pakistan; Department of Nutrition, Exercise and Sports, Faculty of Science, University of Copenhagen, Copenhagen, Denmark; Center for Child Health Research, Tampere University, Faculty of Medicine and Health Technology, Tampere University, Tampere, Finland; Harvard T.H. Chan School of Public Health, Boston, MA, USA; Epicentre, Paris, France; Division of Epidemiology & Biostatistics, University of California, Berkeley, USA; Emergency Nutrition Network, Kidlington, United Kingdom; Brixton Health, Llwyngwril, Gwynedd, Wales, United Kingdom; Francis I. Proctor Foundation, University of California, San Francisco, CA, USA; Friedman School of Nutrition Science and Policy, Tufts University, Boston, MA, USA; Emergency Nutrition Network, Kidlington, United Kingdom; Great Ormond Street Institute of Child Health (ICH), University College London (UCL), London, United Kingdom

**Keywords:** wasting, stunting, concurrent wasting and stunting, research design, child nutrition, mortality risk

## Abstract

Childhood wasting and stunting affect large numbers of children globally. Both are important risk factors for illness and death yet, despite the fact that these conditions can share common risk factors and are often seen in the same child, they are commonly portrayed as relatively distinct manifestations of undernutrition. In 2014, the Wasting and Stunting project was launched by the Emergency Nutrition Network. Its aim was to better understand the complex relationship and associations between wasting and stunting and examine whether current separations that were apparent in approaches to policy, financing, and programs were justified or useful. Based on the project's work, this article aims to bring a wasting and stunting lens to how research is designed and financed in order for the nutrition community to better understand, prevent, and treat child undernutrition. Discussion of lessons learnt focuses on the synergy and temporal relationships between children's weight loss and linear growth faltering, the proximal and distal factors that drive diverse forms of undernutrition, and identifying and targeting people most at risk. Supporting progress in all these areas requires research collaborations across interest groups that highlight the value of research that moves beyond a focus on single forms of undernutrition, and ensures that there is equal attention given to wasting as to other forms of malnutrition, wherever it is present.

See corresponding editorial on page 2634.

## Introduction

The Emergency Nutrition Network (ENN) has been examining the relationship between wasting and stunting for over a decade. Reviews conducted by the ENN in 2013 and 2014 found that wasting was commonly viewed as a short-term, emergency problem, and stunting as a chronic, developmental problem, and that this was reflected in policy, financing models, and program designs (1, 2). These findings were surprising given available evidence suggested that these 2 forms of malnutrition share common risk factors and are often seen in the same child. **Box 1** provides definitions of the undernutrition terms used in this article.

As a result of the findings of these reviews, the Wasting and Stunting project and the Wasting and Stunting Technical Interest Group (WaSt TIG) were initiated by ENN in 2014 with the aim of better understanding the complex relationships and associations between wasting and stunting and of further examining policy, financing, and program approaches to the 2 conditions. From 2014 until 2021, the project has gone through 3 phases and accomplished multiple milestones. During phase I, these milestones included developing an understanding of the relationship between wasting and stunting via a narrative literature review (2), conducting a research prioritization exercise of gaps in knowledge in this area (6), and analyzing 5 high-burden country data sets to estimate the burden of concurrent wasting and stunting (WaSt).

During phases II and III, the focus was on analyses of a much larger pool of secondary data. This enabled the calculation of pooled-prevalence estimates of data from 83 countries of the burden of children who are WaSt (7), examination of how best to identify children who are WaSt and at high risk of dying (8), and exploration of the direct relationship between wasting and stunting in individual children over time (9).

Based on this work, the publication of a policy brief, “Child wasting and stunting: Time to overcome the separation” (10), in 2018 and a *Lancet* viewpoint article (11) in 2019 presented scientific grounds for concluding that the current separation between wasting and stunting in policy, programs, and research is not justified. The articles called for a radical change in how we understand and address childhood wasting and stunting. Phase IV of the project is now ongoing and aims to fill some of the remaining evidence gaps linked to the relationship between wasting and stunting and to translate the implications of findings to date for nutrition policies and programs both at global and country levels.

This article aims to bring a wasting and stunting lens to how research is designed and financed in order for the nutrition community to better understand, prevent, and treat child undernutrition. The strengths and weaknesses of research concerned with undernutrition, as well as opportunities to improve research designs to fill important knowledge gaps, are discussed.

## Lessons learnt

### Identify and target people most at risk of mortality and growth faltering

Experiencing both wasting and stunting at the same time (concurrently) is associated with considerable excess mortality and a risk of death that is similar to or higher than that associated with severe, single-indicator measures of growth failure (e.g., severe underweight and severe wasting) (5, 12). Recent work by the WaSt TIG using data from Senegal has helped to understand the diagnostic criteria needed to identify children at high risk of death, including those with WaSt, who are therefore in need of nutritional support (8). Two measures commonly used at the community level—the weight-for-age z-score (WAZ) and midupper arm circumference (MUAC)—performed well in identifying this high-risk group and were shown to have potential for identifying those children at the highest risk of dying.

BOX 1: A note on classification of undernutrition

The terms wasted and stunted were introduced in the early 1970s by John Waterlow to differentiate, among underweight children, those who had a low weight in relation to their height (wasted) and those who had a low height in relation to their age (stunted).

The terms wasted and stunted are used to classify children by comparing them to a growth reference or standard based on data from a healthy population. Since 2006, the WHO child growth standards have been used for this comparison (3). A wasted child is commonly defined as one whose weight has fallen below 2 SDs or z-scores of the weight of a healthy child of the same height and sex in the growth standard: that is, a child who has a weight-for-height (WFH) value < −2. In children below 24 months, length is measured instead of height and is compared to a weight-for-length standard. A stunted child is commonly defined as one whose height has fallen below 2 SDs or z-scores of the height of a healthy child of the same age and sex in the growth standard: that is, a child who has a height-for-age (HFA) value < −2. A child is classified as severely wasted or severely stunted if they fall below 3 z-scores of the standard (−3 z-scores). Midupper arm circumference (MUAC) cutoffs have also been used to identify wasting based on the evidence that a low MUAC (typically less than 115 mm) identifies children at high risk of death (4). A child who is concurrently wasted and stunted (WaSt) falls below 2 z-scores of the standards for both WFH and HFA.

The terms wasting and stunting refer to a process rather than a category. Stunting refers to a slowing or halting of linear growth (linear growth faltering), and wasting refers to a loss of body weight or ponderal growth in relation to height (ponderal growth faltering). Both these processes can occur during infancy and childhood and also in utero. The use of the term “wasting” to mean “wasted” and “stunting” to mean “stunted,” however, is quite widespread.

Underweight is a term used to describe children with a low weight in relation to their age (weight-for-age) when compared to the WHO 2006 growth standards. This is a composite indicator for wasting and stunting and is used for growth monitoring programs. All children who are WaSt are also underweight (5).

These diagnostic criteria are also appropriate for assessing risks of death in infants under 6 months of age, as well as in older children. A risk of death in infants is traditionally assessed using a weight-for-length z-score (WLZ) for infants less than 6 months, but the WLZ cannot be calculated when length is <45 cm, due to the exclusion of infants <45 cm in length in the 2006 WHO growth standards (3). In a recent study, the use of low birth weight (LBW), low MUAC, and low WAZ values was found to identify high-risk infants under 6 months (13). This has important implications in reaching the most vulnerable infants in a way that does not miss the smallest children with the highest neonatal risk, is practical, and is potentially less open to measurement error than the weight-for-height z-score (WHZ) or WLZ [the measurement of length and height has been shown to be impractical, because it requires at least 2 trained health workers and a height board that is rarely available at the community level (4)]. As such, research that aims to address risks of mortality over time for vulnerable children should utilize and further explore the use of LBW, MUAC, and WAZ values.

There are, however, limits in the use of anthropometry for identifying risks of mortality and growth faltering, because it can conceal crucial physiological variability, connections in pathways, or underlying physiology. We urgently need innovative and early markers to predict, identify, and monitor children at short-term and long-term risks of weight and linear growth faltering. Given that child undernutrition is often established by the time a child is born, we require more research focus on maternal factors that can potentially affect in utero and postnatal child weight and linear growth faltering. Such factors include maternal age, weight, anthropometric status, and education (14), as well as early marriage (occurring at ≤14 years of age) and psychosocial stress (15), from adolescence through pregnancy.

Work by the MAMI (Management of Small Nutritionally At-Risk Infants Under 6 Months and Their Mothers) group has recently outlined a range of assessment and screening tools or indicators to identify small and nutritionally at-risk infants (<6 months of age) and their mothers (16). Most of these tools and indicators are not based on anthropometric measures but on other clinical, social, and environmental factors that are known to influence risk. It is important to better understand the relevance of these factors across different contexts as early markers of wasting and stunting. Other research is building the evidence between other outcomes, such as body composition and functions in the short and long term, and risks for growth faltering and mortality (17). This includes work that examines the relationship between weight and body composition in infants, which may be the product of different processes in pregnancy and that defines the phenotype in relation to fetal growth patterns and events after birth (18).

Age and sex are also important when it comes to identifying risk. As well as the work discussed above that highlights high levels of being wasted and being stunted at birth, recent systematic reviews published by the WaSt TIG highlight population-level data that show that wasting, stunting, and being WaSt are all more prevalent in boys than girls, and that wasting is higher in younger children, while stunting is higher in older children (19, 20). Possible explanatory factors for this are being explored (21). In the case of children with WaSt, the timing of peak prevalence appears between 12 and 30 months with younger children (≥12 and <24 months), with boys being more affected than girls. Recent work suggests that this peak in the prevalence of children with WaSt is largely driven by an increase in stunting up to that age, in combination with more transient wasting, so research that focuses on the process (as discussed above) of linear growth faltering from birth and throughout infancy is key to our understanding of wasting and stunting (22).

### The synergy and temporal relationships between children's weight loss and linear growth faltering

#### Understanding the burden and overlap of wasting and stunting

To better understand the burden of undernutrition, there is a need to systematically report on the combined extent of wasting and stunting in the 0- to 59‐month population, and on the concurrence of stunting and wasting, which carries a mortality risk similar to that of being severely wasted (7). This would help to provide a more complete picture of the scale of these problems and of how the 2 manifestations of undernutrition overlap in different settings. The most recent *Lancet* series on maternal and child undernutrition has now increased focus, compared to previous series, on the associations between wasting and stunting (23). Articles in the series discuss points such as the increased mortality risk for and prevalence of children with WaSt, with the latter both portrayed graphically and also then described within its own narrative section. Other recent work, such as that by Harding et al. (24), also reports on the existence of children with WaSt. Using survey data from several countries in South Asia, they found the prevalence of wasting varied from 9.5% in Afghanistan to 21.0% in India, while the prevalence of stunting in the same sample ranged from 18.0% in the Maldives to 44.4% in Pakistan. Over 5% of Bangladeshi, Indian, and Pakistani children were WaSt, and this was highlighted as requiring urgent attention given the associated high mortality risk. They also found that the underlying determinants of wasting overlapped with those of stunting across these countries, and the authors argued for joint attention to the drivers of wasting and stunting, with a view to better preventing both.

Critically, reporting the numbers of children experiencing wasting or stunting (including the overlap) helps in our understanding of the total burden of undernutrition in children. In a piece written by members of the WaSt TIG for the 2016 Global Nutrition Report, for example, we were able to say that more than a third of children in low- and middle- income countries (LMIC) were affected by either wasting or stunting (25); this was a larger estimate of the burden of undernutrition affecting children under 5 than that using stunting numbers alone.

In order to further understand the burden of undernutrition, we also need to measure and report on the incidence of wasting. Given the transitory nature of wasting in particular, where a child can experience several acute episodes during a year, the use of cross‐sectional data is likely to lead to considerable underestimation of the actual burden. In Mali, Huybregts et al. (26) found that a baseline prevalence of severe acute malnutrition (SAM) of 2.1% to 2.7% in 0- to 18-month-old children translated to an annual SAM incidence of approximately 17.5% in both longitudinal intervention and comparison groups, illustrating the degree to which prevalence data can underestimate the true burden (26). This also means that we are likely to be underestimating the true burden of children with WaSt. Mertens et al. (22) looked at the associations between wasting and stunting to study the cumulative incidence and timing of their overlap. They found that 10.6% of children were WaSt before 2 years of age, and a further 1.5% experienced concurrent severe wasting and stunting, far higher than point prevalence estimates in either the same study or as estimated in cross-sectional surveys. Whilst the measurement and reporting of the incidence rate is clearly valuable, it is acknowledged that the resources and skills needed for such research, which require the collection of longitudinal data (discussed below), are considerably different than those needed for prevalence studies, and that this may be prohibitive if resources are constrained. Recent work has updated incidence correction factors in an attempt to improve program planning when incidence data are not available. This could help to inform the approach to more accurate burden estimations for future work (27).

#### Understanding the process of becoming wasted and stunted

Research has long treated wasting and stunting as separate phenomena that are measured, using anthropometric outcomes, at 1 point in time. Whilst this is important for identifying those most at risk of mortality (discussed above), it hinders our understanding of the relationship between the 2 conditions or processes and, ultimately, of the most effective responses (11, 28). There is increasing recognition, however, that it is important to consider the process that leads to an individual becoming wasted or stunted: that is, the process of wasting and stunting. The work of the WaSt TIG has found that wasting and stunting are in fact frequently overlapping processes, sometimes occurring at the same time in the same child and often, but not always, influenced by similar risk factors, including poverty, the mother's nutrition and health status, food insecurity, the infectious disease burden, and water, sanitation, and hygiene conditions (9, 29). If studies only look for the relationship between being wasted and stunted at 1 moment in time, they are likely to miss the multitude of associations between outcomes at birth; periods of wasting (or weight loss) or weight gain, including during treatment; and the slowing, halting, or acceleration of linear growth. In the latest *Lancet* (2021) maternal and child undernutrition update series, Victora et al. (23) presented the distribution of height-for-age z-score (HAZ) and WHZ curves from the Demographic and Health Surveys (DHS) included in the paper's analysis, and examined how these compared to the WHO (2006) growth standards. In the paper, the DHS surveys had HAZ and WHZ distributions that were both shifted to the left of the growth standard curves. This meant that populations represented by the DHS surveys not only had increased proportions of stunted and wasted children compared to well-nourished populations, but that the whole population had lower WHZ and HAZ values than if they had been well nourished. This reinforces a need for research to focus on better understanding the processes of wasting and stunting and how many children grow below their potential, because these aspects, as well as being wasted or stunted, have implications for future growth and health outcomes (9).

### Diverse pathways that drive different forms of undernutrition

Stunting and wasting share many causal pathways (30) yet, as outlined in our 2019 Viewpoint article (11) and as discussed above, a reliance on anthropometric cutoffs to define each condition gives an incomplete picture of these causal pathways and the different risks associated with them. To better understand this for different children in different contexts, we need more data, not only on how anthropometric measures change over time but also on the interlinked and dynamic biological processes and pathways that underlie the spectrum of child weight loss and linear growth faltering in different settings. In particular, there is a need to better understand how these processes differ for infants born stunted and/or wasted compared with infants that develop wasting and/or stunting around 3 to 4 months of age (9, 31). This would enable a deeper analysis of relationships between the causes and timing of child weight and linear growth faltering.

High prevalences of wasting (or being wasted) and stunting (or being stunted) are present in infants at birth. Recent estimates from large, longitudinal cohorts suggest that 13% of infants in LMIC are born stunted, rising to 20% in South Asia (32), and 12% of infants are born wasted, rising to 19% in South Asia (22). Whilst newborn stunting and wasting are products of different processes in pregnancy, including nutritional deficits, placental pathology, or maternal disease, they are also influenced by gestational age at birth. For example, some infants classified as wasted or stunted could be misclassified if they were born preterm and are a healthy size for their gestational age (33). Z-scores can be corrected for gestational age using the INTERGROWTH-21st standards (33), which reduces the prevalence of growth failure at birth (34) and is especially important in LMIC, where preterm birth rates are high (35). However, fetal ultrasound, the gold-standard method for determining gestational age, can be challenging and expensive to implement in studies of child growth in rural or low-resource settings, as it requires both an ultrasound machine and enrolling pregnant women in their first trimester. There are alternative, less accurate methods based on newborn examinations or recalls of the date of last menstruation, but further improvements in low-cost gestational age assessment methods and corrections of z-scores for gestational age would reduce infant growth failure misclassifications (36–38).

Mertens et al. (14) have also shown that maternal and child characteristics at birth may account for the largest attributable differences in postnatal growth, with preterm birth, birth anthropometry, maternal anthropometry, and maternal education among the top predictors of length-for-age and weight-for-length at 24 months. In the same work, all measures of early child weight and linear growth faltering (before 6 months of age) were significantly associated with later, more serious growth faltering, with wasting indicators among the strongest of predictors. There are also other causes of later growth faltering with, for example, characteristics of the child's household environment being additional determinants of growth faltering after 6 months of age, and factors such as periods of rapid weight gain during wasting treatment being associated with deterioration in HAZ (39). Seasonality is also an important and complex factor that impacts nutritional stress. Mertens et al. (22) have recently shown that the season in which a child is born strongly predicts their birthweight and subsequent linear growth and weight gain. The season of birth could also reflect the season of conception, including the conditions of the mother at the time of conception and her health and well-being through pregnancy.

Work in Nepal has shown that seasonality has different associations with a wide range of factors that impact early nutrition, such as maternal diet and food insecurity, maternal BMI, and neonatal anthropometry (40). Seasonality is also associated with wasting in older children (>6 months of age), and encompasses a range of factors that are influenced by season and are known to be important for child nutrition after infancy, such as dietary diversity, nutrient consumption, micronutrient status, maternal and child morbidity indicators, pathogen-specific infections, subclinical inflammation, and intestinal dysfunction (40, 41).

Future research needs to unpack more precisely what seasonality represents as a proxy or marker for multiple environmental exposures that impact maternal and child nutrition in different contexts. These include the infectious disease burden; food prices, scarcity, or diversity; direct effects of temperature on fetal growth; and maternal physical activity and energy balance. To do this, as discussed in recent work by Marshak et al. (42), there is a need to improve both the design and analysis of nutrition seasonality research. In particular, we need a better understanding of the main seasonal drivers of poor fetal and child growth in different contexts and how we can best mitigate the negative effects they are having.

### The value of longitudinal data

Work supported by the WaSt TIG and others that has drawn on longitudinal data has been valuable in better understanding the relationship between wasting and stunting, especially whereby episodes of wasting contribute to later episodes of stunting (29). Longitudinal data from the Gambia showed that being wasted in the previous 3 months increased the odds of being stunted by 3.2 (95% CI, 2.7–3.9) (9). A multicountry, longitudinal analysis showed that persistent wasting (defined as an individual being wasted in >50% of the measurements) from birth to 6 months was strongly associated with incident stunting at older (>6 months) ages. It also showed that height velocity is slowest among children with the lowest WHZ prior to the 3-month period at which height velocity is measured (22). Both studies indicate a time-lagged effect whereby wasting is followed by stunting. This work suggests that preventing wasting, as well as identifying and treating it early, may be important for preventing stunting.

Whilst some data exist, there is a need for more robust longitudinal panel data and tracking of growth over time, including sufficient data collection frequency, improved data quality, the capture of more appropriate exposures (including prenatal factors where possible), and the calculation of growth velocity as an outcome. This would help to improve our understanding of the incidence of wasting, the association between wasting and stunting at the population level, and the pathways and risk factors associated with becoming stunted or wasted. Additionally, it could help to elucidate how the process of stunting and wasting is affected by temporal factors, such as season, and by geography. More longitudinal data are also needed to fill particular research gaps linked to the relationship between being WaSt and mortality. For example, we do not yet know whether it makes a difference to mortality risk being WaSt occurs in a child who has been stunted for months or years then experiences rapid weight loss, compared to a persistently wasted child who slowly becomes stunted through limited linear growth. Nor do we know whether the heightened mortality risk is limited to those experiencing a tight time frame of concurrence (i.e., restricted to simultaneously experiencing both conditions), or whether episodes of wasting and stunting falling consecutively but not necessarily simultaneously also confers an elevated mortality risk. Whilst new data could help to fill these gaps, existing longitudinal data sets, reanalyzed through a wasting and stunting lens, might be equally as valuable.

## Implications for Future Nutrition Research

Many recent leading nutrition publications, strategies, and plans call for more and improved research to support the delivery of impactful actions for the prevention and treatment of child undernutrition. “No Time to Waste,” UNICEF's approach for the prevention, early detection, and treatment of wasting in early childhood, for example, highlights the need to unpack the context-specific determinants and drivers of wasting in order to develop appropriate preventive interventions that include the elements needed to address wasting alongside stunting (43). The UN's Global Action Plan on child wasting summarizes key research priorities that also reflect many of the issues raised above, including the need for improved identification of nutritional risks at individual and community levels, increased focus on maternal health and infant nutrition, and the importance of the early stages of life in determining later experiences of wasting (44). Other recent work, led by members of the WaSt TIG and others, such as the Child Health & Nutrition Research Initiative (CHNRI) prioritization exercises on the prevention of child wasting (6) and the treatment of child wasting (45), also discuss many of the same issues. Specific suggestions for future research priorities can be found in the CHNRI reports, as well as in dedicated sections on the research priorities emerging from recent systematic reviews (20).

In order to respond to these calls, we need to improve several aspects of the way we design and conduct nutritional research. This paper has discussed several key areas that future nutrition research should prioritize, informed by several years of work by the WaSt TIG:

- Systematically report on the combined extent of wasting and stunting in the 0‐ to 59‐month population and on the concurrence of stunting and wasting, including better measurement and reporting of the incidence of wasting.
- Increase focus on the process of stunting and wasting rather than the outcomes themselves, including the composite stresses that impact mothers, especially during pregnancy, and the impacts of addressing 1 outcome (e.g., wasting) on the other (e.g., stunting).
- Develop a better understanding of the risk factors that drive child weight loss and linear growth faltering in different contexts at different times. This is critical for understanding and interrupting the process of becoming stunted and wasted. This includes the need to better understand how these factors differ for infants born stunted and/or wasted compared with infants that develop wasting and/or stunting around 3 to 4 months of age. More research therefore needs to include the preconception, in utero, and infancy periods, and studies should focus on both the mother and child.
- The importance of season is paramount to better understanding the drivers of infant and child weight and linear growth faltering. It relates to variability in factors that underlie early nutritional stress, such as women's agricultural workload, risks of maternal or child infections, availability of harvest for consumption, and seasonality of wages. To better understand the main seasonal drivers of poor fetal and child growth in different contexts, and how we can best mitigate the negative effects they are having, we need to improve both the design and analysis of nutrition seasonality research. Age and sex have also been highlighted as factors that drive vulnerability to undernutrition. As such, there is a need to power studies sufficiently to enable stratification by age and sex categories in order for important differences in risk between groups to be highlighted more widely.
- To enable this, we need more robust longitudinal panel data and to track growth over time, starting with measurements on the mother and not just the child. The calculation of growth velocity as an outcome, as well as the estimation of the prevalence of undernutrition, would support a better understanding of those growth trajectories prior to wasting, stunting, and being WaSt that indicate the highest risk of mortality. Whilst the collection of new data would help to fill these knowledge gaps, existing longitudinal data sets, reanalyzed with a wasting and stunting lens, might be as equally valuable. There is also a need to include gestational age and body composition measurements in studies that are looking at changes in nutritional status over time.
- Use diagnostic criteria that are known to better identify those infants and children at high risk of death. This may have implications for treatment that need to be explored. Work by the WaSt TIG has found that mortality risks differ according to the combination of deficits (low MUAC, low WAZ, or both) that a child experiences, and suggests that different levels of treatment intensity may be appropriate based on the deficit(s) present (Khara T, Myatt M, Sadler K, Bahwere P, Berkely JA, Black R, Boyd EM, Garenne M, Isanaka S, Lelijveld N, et al., unpublished results, 2022). This is important to consider given both the increase in caseloads likely if a combined case definition (e.g., low MUAC and low WAZ) were to be adopted by treatment programs and the need to control program costs and resource implications.
- Develop more innovative and early markers to predict, identify, and monitor children at short-term and long-term risks of weight and linear growth faltering. This includes markers that can identify risks for inadequate fetal growth in pregnancy, such as nutritional deficits, placental pathology, or maternal disease, or physical or psychosocial threats to women.

Supporting progress in all these areas requires research collaborations across interest groups that move beyond a focus on single forms of undernutrition and ensures that there is equal attention given to wasting, wherever it is present, as to other forms of malnutrition. Given the overlap in determinants of stunting and wasting, there are also considerable learning opportunities provided by robust program impact evaluations that examine impacts upon both stunting and wasting across both development and crisis settings (11). Donors, international and national organizations, governments, and research institutions should use the lessons from the WaSt TIG presented here to inform more coherent research and evaluation investments in this critically important area.

## Notes

The work reported here was made possible by the generous support of the American people through the United States Agency for International Development (USAID) and Irish Aid, Grant/Award Number HQPU/2021/ENN.

Author disclosures: The authors report no conflicts of interest.

Perspective articles allow authors to take a position on a topic of current major importance or controversy in the field of nutrition. As such, these articles could include statements based on author opinions or points of view. Opinions expressed in Perspective articles are those of the author and are not attributable to the funder(s) or the sponsor(s) or the publisher, Editor, or Editorial Board of *The Journal of Nutrition*. Individuals with different positions on the topic of a Perspective are invited to submit their comments in the form of a Perspectives article or in a Letter to the Editor.

Abbreviations used: CHNRI, Child Health & Nutrition Research Initiative; DHS, Demographic and Health Survey; ENN, Emergency Nutrition Network; HAZ, height-for-age z-score; HFA, height-for-age; LBW, low birthweight; LMIC, low- and middle- income countries; MUAC, midupper arm circumference; SAM, severe acute malnutrition; WaSt, concurrently wasted and stunted; WaSt TIG, Wasting and Stunting Technical Interest Group; WAZ, weight-for-age z-score; WFH, weight-for-height; WHZ, weight-for-height z-score; WLZ, weight for length z-score.
